# AI-driven multimodal colorimetric analytics for biomedical and behavioral health diagnostics

**DOI:** 10.1016/j.csbj.2025.05.015

**Published:** 2025-05-28

**Authors:** Desta Haileselassie Hagos, Saurav Keshari Aryal, Patrick Ymele-Leki, Legand L. Burge

**Affiliations:** a Howard University, Department of Electrical Engineering and Computer Science, 2400 Sixth Street NW, Washington DC, 20059, DC, USA; b Howard University, Department of Chemical Engineering, 2400 Sixth Street NW, Washington DC, 20059, DC, USA

**Keywords:** AI model, Biomedical, Multimodal, Colorimetric

## Abstract

The exponential growth of multi-scale biomedical and behavioral data introduces both challenges and opportunities for -driven analytics. Effectively managing the complexity and variability of these data sources requires advanced computational techniques for accurate interpretation and robust decision-making. Integrating  with colorimetric biosensing and multimodal data fusion offers scalable solutions that can improve diagnostic accuracy, enable early disease detection, and support personalized medicine. This work explores mobile-based colorimetry, an -driven approach that uses image processing and  to detect colorimetric changes in chemical and biological solutions. We propose a modular conceptual framework that integrates mobile-based colorimetry with multimodal biomedical data, such as clinical, imaging, and environmental datasets, to develop scalable, low-cost tools for predictive modeling, real-time health monitoring, and personalized diagnostics. We review recent advancements in -enabled colorimetric analysis and multimodal data fusion for healthcare applications, emphasizing innovations in -assisted biosensors, -driven biomedical imaging, and multimodal fusion techniques. In addition, we highlight the need for robust data management systems and interpretable AI/ML models to ensure security, privacy, and reliability in biomedical and behavioral research. This work also highlights practical directions for improving diagnostic accuracy and accessibility, particularly in resource-limited settings.

## Graphical abstract

**Figure:**
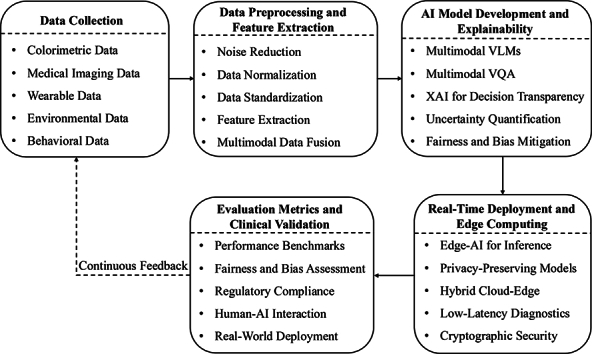

## 1 Introduction

Advancements in  and  are transforming biomedical and behavioral health research by enabling more accurate and efficient health monitoring systems to analyze complex biomedical data. From medical image analysis to clinical note interpretation, AI tools now support many areas of healthcare [1], [2]. The growing availability of multimodal data from colorimetric biosensors [3], wearable devices [4],  [5], and mobile sensing platforms [6] offers opportunities for early disease detection, personalized treatment, and real-time health monitoring. As biomedical and behavioral health research is increasingly relying on data from clinical records, imaging, wearable devices, personal devices, and even social and environmental sources, making sense of all this information in real time is a growing challenge. These data are often complex, varied, and scattered across different systems, making integration and interpretation difficult. Although mobile sensing and multimodal diagnostics hold promise, many current solutions are incomplete. To fully benefit from this growing data landscape, we need smarter and more efficient  tools that can simplify, combine, and interpret data from different sources to help researchers and clinicians make faster and more accurate decisions in health research and practice.

To address these challenges, -driven colorimetric biosensing has emerged as a promising technique for disease detection, environmental monitoring, and personalized diagnostics [7], [8]. By leveraging  algorithms, these systems can interpret spectral changes and biomarker variations with high sensitivity, enabling rapid and non-invasive analysis. In addition, the fusion of multimodal data, integrating biosensor readings, imaging, and clinical data, has become a critical research area, significantly improving disease prediction, treatment personalization, and real-time monitoring systems [9], [10], [11], [12]. As mobile technologies advance, mobile-based colorimetry has gained attention as a scalable and cost-effective solution for biomedical and behavioral research [13], [14]. When combined with AI/ML, mobile-based colorimetric systems can improve data accessibility, facilitate remote diagnostics, and enable real-time health monitoring. These advances open new opportunities for transformative analytics, bridging the gap between laboratory-grade accuracy and real-world applications in resource-limited settings.

Focusing on this growing intersection of mobile sensing and health analytics, we explore the role of  and  in advancing biomedical and behavioral health research, particularly mobile-based colorimetry as a scalable and cost-effective method for detecting colorimetric changes in chemical and biological solutions. Mobile-based colorimetry uses smartphone cameras and AI algorithms to measure colorimetric changes, offering a low-cost, accessible, and non-invasive solution for disease detection, personalized diagnostics, and environmental monitoring [15], [16]. In the context of behavioral health, this technology can be applied to measure biomarkers associated with stress, mental health, or substance use [17], [18], providing valuable insights into behavioral health outcomes [19]. For example, colorimetric biosensors could be used to detect stress-related biomarkers, such as cortisol or other metabolites, typically measured in saliva, sweat, or urine, offering potential for real-time monitoring and early intervention in non-clinical settings [17], [20], [21]. Moreover, multimodal data fusion techniques allow the integration of colorimetric readings with behavioral indicators, such as heart rate variability, sleep patterns, and neurophysiological signals [22], [23], offering new opportunities for real-time assessment and monitoring of mental health conditions. By integrating mobile-based colorimetry with multimodal data, such as clinical records, wearable device data, and environmental factors, researchers can develop innovative models to study the complex interplay between biological and behavioral health [24]. This approach improves our understanding of health and disease and addresses critical challenges such as noisy and limited data records, particularly in resource-limited settings [8].

This review focuses on one main question: *How can AI-driven systems be designed to support integrated, real-time diagnostics using colorimetric biosensing and multimodal biomedical data in resource-constrained settings*? Existing reviews typically focus on individual components, such as biosensor design, wearable data modeling, and explainable AI, but rarely integrate these dimensions into a unified diagnostic framework. To address this gap, we propose a conceptual architecture that combines mobile-based colorimetric biosensing with multimodal data fusion and behavioral context, organized into five key dimensions: (*i*) mobile biosensing technologies, (*ii*) cross-modal integration techniques, (*iii*) behavioral signal modeling, (*iv*) interpretability frameworks, and (*v*) edge-compatible deployment. This paper reviews recent advances in each domain and highlights practical insights that support the development of real-time, scalable, and interpretable diagnostic AI systems.

To demonstrate the practical impact of these integrated systems, we highlight the potential of mobile-based platforms that use image processing and  techniques to transform biomedical and behavioral research. These systems enable efficient data reduction, improved knowledge representation, and better interpretability of  models in biomedical contexts. In behavioral research, they complement traditional methods by providing objective, quantifiable data on biomarkers associated with psychological states. Furthermore, we propose a framework for integrating mobile-based colorimetry with multimodal data, such as clinical records, wearable device data, and environmental factors, to improve predictive modeling, real-time health monitoring, and personalized diagnostics. This integrated approach not only advances biomedical research, but also provides new tools to understand the biological mechanisms of behavioral health. By reviewing recent advances in -enabled colorimetric biosensing, -based biosensors, and multimodal data integration, we provide a comprehensive perspective on how  is transforming biomedical and behavioral health research. We also highlight the importance of robust data management strategies and interpretable models to address the challenges related to privacy, security, and diagnostic reliability. Our goal is to provide a useful roadmap for developing diagnostic tools that are not only scalable, but also clinically relevant and responsive to real-world constraints.

## 2 Background and related work

Mobile-based colorimetric biosensing and multimodal biomedical data fusion are emerging as valuable diagnostic tools, particularly in low-resource and decentralized healthcare settings. These approaches aim to bridge the gap between laboratory-grade analysis and portable, user-friendly diagnostics. Colorimetric biosensing relies on visible color changes to quantify chemical or biological markers. Recent studies have used smartphone cameras and image processing algorithms to analyze samples such as saliva, urine, and blood [7], [13]. These approaches support low-cost, real-time detection of biomarkers for conditions such as glucose monitoring, infectious diseases, and environmental toxins. Advances in machine-readable lateral flow assays and optical signal processing have improved the accessibility of colorimetric diagnostics beyond traditional laboratory settings.

In parallel, multimodal data fusion has emerged as a powerful technique to integrate heterogeneous biomedical information, including clinical records, wearable sensor data, medical imaging, and behavioral signals, into unified predictive models. Previous studies have shown that combining these diverse data types can improve diagnostic accuracy and support more personalized care [10], [11]. Common strategies include feature-level fusion, hybrid modeling, and cross-modal attention, all of which help to uncover meaningful relationships across modalities. However, key challenges remain, particularly in terms of data alignment, handling missing or noisy input, and maintaining model interpretability. Although mobile colorimetry and multimodal data fusion have shown promise independently, few studies have explored their integration into a unified diagnostic framework, especially in the context of behavioral health. Existing research often targets specific disease conditions or focuses on technical validation without addressing the broader need for accessible, scalable platforms that support real-time and interpretable analysis.

This work builds on these foundations by proposing a conceptual framework that combines mobile-based colorimetric detection with heterogeneous biomedical data sources. The goal is to address current limitations and enable more practical, personalized, and portable diagnostic solutions. Looking through the previous studies included above, we noticed three common patterns. First, most existing papers tend to focus on a single area, such as biosensing, data integration, or behavioral health, without connecting them. Second, few studies have addressed both interpretability and real-time performance together, although both are essential for clinical use. Third, practical challenges such as model deployment on edge devices, explainability, or preserving data privacy are often overlooked. These gaps have helped shape our proposed modular framework, which is designed to connect these fragmented efforts into a unified and deployable solution. Despite the growing interest in AI-driven colorimetric sensing, more research is needed to standardize evaluation metrics and expand its use in areas such as behavioral health and point-of-care diagnostics in low-resource settings.

## 3 Motivation

Despite significant advancements in  and biosensing technologies, effectively integrating  with colorimetric analysis and multimodal healthcare data remains an ongoing challenge [7], [8]. Although mobile-based colorimetry is a scalable and cost-effective approach to detect colorimetric changes in chemical and biological solutions, it has not been fully utilized in real-world biomedical applications. The fusion of mobile-based colorimetry with multimodal data introduces challenges in data heterogeneity, alignment, feature extraction, and real-time interpretability, which are critical challenges to developing robust -driven diagnostic models. Addressing these limitations is important for improving the integration of multimodal biomedical data and enabling advancements in personalized diagnostics and health monitoring. Traditional disease detection and biomarker identification methods often rely on manual or semi-automated techniques, which are time-consuming, prone to human error, and lack scalability. While -driven approaches offer the potential to automate processes and improve diagnostic accuracy, several key challenges remain, including data heterogeneity, model generalization, seamless data integration [25], [26], [27], and explainability [28], [29]. Furthermore, the variability in biosensor data across different devices, settings, and populations further complicates the robustness of the model and the real-world deployment.

Recent advances have explored specific biomarkers for behavioral health diagnostics, especially those accessible using mobile or wearable platforms. In behavioral health applications, biomarkers such as cortisol, dopamine, serotonin, and inflammatory cytokines are increasingly being used to assess psychological stress and mood disorders [30]. Cortisol is commonly measured in saliva using colorimetric assays, with recent developments enabling smartphone-based detection using lateral flow immunoassays [31]. In addition, metabolites such as alpha-amylase and interleukin-6 (IL-6) are being explored for mobile sensing applications [32], [33]. By combining biochemical signals with wearable-derived metrics like  and sleep quality, AI models enable a more comprehensive and interpretable analysis of behavioral health outcomes. Our framework supports the integration of these biomarkers into interpretable diagnostic pipelines to monitor stress and mental health.

A critical gap in existing research is the lack of  models capable of efficiently processing, integrating, and interpreting multimodal biomedical data. The fusion of colorimetric biosensing with clinical, imaging, and environmental data presents unique opportunities to improve diagnostic precision and enable real-time, personalized healthcare solutions. However, this integration requires new  methodologies capable of handling data misalignment, optimizing feature extraction, and advancing cross-domain learning techniques. This work is motivated by the need to bridge the gap between AI methodologies and real-world biomedical and behavioral health applications. Through an in-depth analysis of the latest advancements, we identify emerging trends, highlight challenges, and propose research opportunities to develop robust, interpretable, and scalable AI models in both biomedical and behavioral health research [8], [28]. AI is increasingly being applied to behavioral health research [34], with applications spanning early diagnosis [35], personalized treatment [36], [37], and improved patient engagement [38]. Our goal is to provide researchers and practitioners with actionable insights to advance AI-driven methodologies and improve the clinical utility of AI-driven health monitoring and diagnostic systems.

Table 1 presents a comparative overview of recent AI-enabled colorimetric biosensing platforms, listing their targeted analytes, AI methodologies, and key limitations. We did not include performance metrics due to inconsistent reporting across studies (e.g., varying validation protocols) and the use of review articles that summarize multiple systems without verifiable experimental benchmarks. Instead, the focus is on the methods used and the range of applications. Table 2 categorizes relevant studies based on the type of device integration, wearable, smartphone-based, or microfluidic, to reflect the different ways these technologies are implemented and used in practice across platforms. These two tables provide a clear view of the current technological landscape and help contextualize the need for robust, interpretable, and deployable AI-driven colorimetric sensing systems.

**Table 1:** Summary of representative AI-based colorimetric sensing platforms: Target Analytes, AI Methods, and Key Limitations.

Study	Target Analyte(s)	AI/ML Method(s)	Key Limitations
[3]	Tear biomarkers (e.g., glucose, lactate), Vitamin C, Proteins, (pH),	Hybrid multichannel CNN-GRU (e.g., 1D-CNN-GRU for pH prediction, 3D-CNN-GRU for vitamin C and proteins, and 4D-CNN-GRU for color temperature correction)	Limited transparency, scalability and generalization, Lighting conditions and pH variability significantly affect raw colorimetric readings
[7]	Multiple biomarkers (e.g., glucose, saliva cortisol)	Various deep learning and classical ML techniques such as CNNs, random forests, and k-means	Integration challenges, data variability, real-time application gaps
[8]	Multiple cancer biomarkers (e.g., miRNA-21, HER2, glucose, exhaled breath volatile organic compounds (VOCs))	Various AI/ML techniques including CNNs, neural networks, and random forests for pattern recognition and biomarker detection	Data scarcity, limited clinical translation, limited generalizability, sensor durability issues
[12]	Cancer biomarkers	Various AI/ML classifiers such as SVM, Random Forest, CNN, RNN	Lack of clinical validation, limited generalizability
[15]	Pathogenic bacteria (Hyaluronidase (HAase))	CNN, YOLOv5	Limited generalization across bacteria types, real-world deployment, model transparency
[22]	Mental health signals (stress, anxiety), heart rate, heart rate variability (HRV), electrodermal activity (EDA)/galvanic skin response (GSR), skin temperature (ST), respiratory rate	Various AI/ML techniques and adaptive, personalized models, including Random Forests, SVM, KNN, CNN, logistic regression, and localized learning strategies	Lack of explainability, bias in mental health data, limited population diversity, imbalanced datasets
[23]	Biochemical and chemical analytes (e.g., glucose, lactate, cortisol, uric acid, creatinine, pH, , , , cytokines, insulin, glucagon, bilirubin, drugs)	Multimodal data fusion and pattern recognition, clustering, regression, and feature extraction techniques	Complexity of data fusion challenges, clinical interpretability concerns, user variability, sensor biofouling
[24]	Multiple physiological and biochemical signals (e.g., sweat, pulse, neurotransmitters, VOCs)	General ML and AI integration with multimodal sensor systems; no specific algorithm detailed	Lacks algorithmic specifics; focuses on broad integration trends rather than targeted colorimetric applications

**Table 2:** AI-enabled colorimetric biosensing studies categorized by device type.

Study	Wearable	Smartphone-Based	Microfluidic
[3]	✓	–	✓
[8]	–	–	✓
[12]	–	✓	✓
[13]	✓	✓	–
[14]	–	✓	✓
[15]	–	✓	✓
[16]	–	✓	–
[18]	✓	–	–
[19]	✓	–	–
[20]	✓	–	✓
[21]	–	–	✓
[22]	✓	–	–
[23]	✓	–	–
[30]	✓	–	–
[31]	–	✓	–
[32]	–	–	✓

### 3.1 Literature review methodology

To ground our proposed framework within the current research landscape, we conducted a targeted literature review using a structured search strategy focused on AI-driven colorimetric sensing, multimodal biomedical data fusion, and behavioral health diagnostics. We examined articles published between 2014 and 2025, with the exception of some related work studies from previous years. We searched key scientific databases, such as IEEE Xplore, Scopus, Google Scholar, and PubMed, as well as leading AI venues, including NeurIPS, ICLR, and Nature Machine Intelligence. The key search terms included a combination of the following: “mobile-based colorimetry”, “AI biosensing”, “multimodal data fusion”, “behavioral health diagnostics”, “AI in biomedical imaging”, and “explainable AI in healthcare”. We included studies that (*1*) presented original research on AI-driven biosensing or multimodal analytics in biomedical or behavioral health contexts; (*2*) employed mobile or low-cost diagnostic technologies; (*3*) focused on colorimetric or biosensor-based data collection, or (*4*) addressed challenges in data integration, model interpretability, or real-time deployment. We excluded review papers (except those cited for background), opinion pieces, editorials, and studies that lacked technical rigor or clinical relevance. The resulting review identified critical trends and technology gaps in the literature, which informed the structure of our key challenge analysis (Section 4) and directly motivated the design opportunities of our proposed framework (Section 5).

Table 3 summarizes 15 representative studies selected based on our review criteria, covering recent and foundational advances in mobile biosensing [7], [8], [12], multimodal biomedical data fusion [9], [10], [39], behavioral health diagnostics [34], [35], explainable AI [29], fairness and bias mitigation [40], and privacy-preserving ML [41], [42]. Additional studies address data integration [26] and IoT-enabled sensing platforms [6]. These works highlight critical gaps in the integration of real-time sensing, multimodal fusion, and interpretable AI for scalable and personalized diagnostics.

**Table 3:** Summary of representative studies in AI-driven biosensing, multimodal data fusion, behavioral health, and clinical ML.

Study	Domain	Data Types	AI Techniques	Key Contributions	Limitations
[7]	Biosensing	Colorimetric	ML, Image Processing	Reviewed challenges and innovations in AI-based point-of-care biosensing	Lacks integration with multimodal or contextual data
[8]	Biosensing	Biomarkers, Sensor Signals	Supervised ML, Classification	Discussed AI-enabled biosensors for early detection and monitoring	Focused on device-level performance; lacks systems integration
[9]	Data Fusion	Imaging, Clinical, Omics	Deep Fusion Networks, Transformers	Surveyed DL techniques for biomedical data fusion	Focused on biological data; lacks behavioral diagnostics
[10]	Multimodal	Clinical, Imaging	DL, Fusion	Overview of multimodal ML for precision health	Limited coverage of low-cost sensing
[11]	Multimodal	Imaging, Genomics	Deep Fusion Networks	Multimodal DL for cancer biomarker discovery	Biomedical focus, no behavioral angle
[12]	Biosensing	Colorimetric	ML for signal analysis	Demonstrated ML-enhanced biomarker detection	No integration with multimodal data
[26]	Precision Medicine	Clinical, Genomic	Integration Frameworks, Data Cleaning	Outlined data integration challenges for ML in precision medicine	No focus on colorimetry, mobility, or behavioral data
[29]	XAI	Clinical, Imaging	Causal Modeling, SHAP, LIME	Offered a multidisciplinary perspective on AI interpretability in healthcare	Theoretical; lacks discussion of real-time or sensor-based systems
[34]	Mental Health AI	Clinical, Behavioral, Wearables	ML, Predictive Analytics	Explored applications and barriers of AI in mental health care	Conceptual framework; limited methodological depth
[35]	Mental Health AI	Behavioral, Social Media, EHR	Overview, ML, NLP	Surveyed AI applications across psychiatric and cognitive health domains	Limited coverage of sensor-based or real-time diagnostics
[39]	Biomedical AI	Imaging, Clinical, Sensors	Multimodal ML, Cross-modal Fusion	Comprehensive overview of multimodal biomedical AI strategies	Lacks focus on mobile-based sensing or colorimetric analytics
[41]	Privacy-Preserving	Federated EHR, IoT	Federated Learning, Secure AI	Reviewed federated learning methods for distributed and secured medical data	Lacks connection to biosensing or real-time mobile sensing or colorimetry
[42]	Secure Computation	Genomic, Clinical	Homomorphic Encryption, Federated Analytics	Proposed scalable, privacy-preserving analytics using multiparty computation	High computational overhead; not yet adapted to mobile devices
[6]	Internet of Things	Environmental, Wearables	IoT Architectures, Sensor Networks	Provided early vision of IoT for health monitoring and smart sensing	Predates modern ML; lacks AI and diagnostic focus
[40]	Bias Mitigation	Clinical, EHR	Adversarial Training	Proposed framework for reducing algorithmic bias in clinical models	Focused on tabular models; no mobile or sensor applications

### 3.2 Scope and objectives

This paper aims to bridge critical gaps in AI-driven biomedical diagnostics by unifying three key domains that have traditionally been addressed in isolation: mobile-based colorimetric biosensing, multimodal integration of biomedical data, and behavioral health analytics. Although previous studies have made significant contributions in each of these areas, most have focused narrowly on individual components, for example, low-cost biosensing [7], [8], large-scale multimodal fusion [39], [9], or behavioral health modeling [34], [35] without offering a comprehensive, real-time, and interpretable solution that operates effectively in decentralized or low-resource environments.

As detailed in Table 3, we reviewed 15 key studies that represent fundamental and recent advances in biosensing, multimodal integration, and AI for biomedical applications, highlighting their specific contributions and limitations in mobile diagnostics, behavioral health, explainability, and privacy-preserving computation. To further clarify the scope and novelty of this work, Table 4 compares these studies against five core dimensions central to real-world deployment of diagnostic AI: mobile biosensing, multimodal integration, relevance of behavioral health, model interpretability, and edge-compatible (real-time) deployment. Unlike previous studies that treat these areas in isolation, this paper presents a unified conceptual modular framework that integrates mobile colorimetric biosensing with multimodal biomedical and behavioral data streams. Our approach emphasizes not only diagnostic accuracy but also practical considerations such as real-time processing, model transparency, and privacy-aware inference, making it particularly suited for decentralized, resource-limited, or wearable-compatible applications. Building on the identified gaps in the existing literature and current technological limitations, this work aims to:

• Summarize recent advancements in mobile-based colorimetric biosensing and multimodal data fusion, and identify current limitations.
• Analyze the challenges and limitations of current methodologies in biomedical  applications.
• Propose research directions to advance AI-driven biomedical and behavioral health diagnostics, with a focus on model interpretability, real-time processing, and multimodal data integration.

**Table 4:** Comparison of core capabilities across representative studies in AI-driven biomedical analytics. Each study is evaluated along five key dimensions: mobile biosensing, multimodal fusion, behavioral health relevance, model interpretability, and real-time deployment. Our proposed framework uniquely integrates all five elements into a unified and modular system.

Study	Mobile Biosensing	Multimodal Integration	Behavioral Health	Interpretable AI	Real-Time
[7]	✓	–	–	–	–
[8]	✓	–	–	–	–
[9]	–	✓	–	–	–
[10]	–	✓	✓	–	–
[11]	–	✓	–	–	–
[12]	✓	–	–	–	–
[26]	–	✓	–	–	–
[29]	–	–	–	✓	–
[34]	–	–	✓	–	–
[35]	–	–	✓	–	–
[39]	–	✓	–	–	–
[40]	–	–	–	✓	–
[41]	–	–	–	–	✓
[42]	–	–	–	–	✓
[6]	–	–	–	–	✓

**This Paper**	✓	✓	✓	✓	✓

The scope and objectives of this work provide a foundation for addressing key challenges in biomedical and behavioral health AI, which are explored in the next section.

## 4 Challenges in biomedical and behavioral research

While  and  have made significant progress and have transformative potential for biomedical and behavioral research, substantial challenges limit their widespread adoption and clinical deployment. These challenges come from the complexity of working with multimodal data [9], [10], [43], the need for interpretable models [29], [44], [45], ethical concerns [46], [47], and computational limitations in real-time healthcare settings [48]. Addressing these limitations is critical to realizing the full potential of AI-powered methodologies, which can improve diagnostic accuracy, enable personalized healthcare, and support data-driven decision-making. By addressing these challenges, AI-powered biomedical research can drive major improvements in healthcare outcomes, early disease detection, and the development of personalized medicine. In particular, AI-driven colorimetric analytics and multimodal data fusion offer promising pathways to revolutionizing biomedical and behavioral health diagnostics. Successfully addressing key issues such as data heterogeneity, model interpretability, bias, computational efficiency, and regulatory compliance is essential for the safe and effective deployment of AI-driven solutions in real-world clinical settings. This section outlines the main challenges in integrating  with biomedical and behavioral data, while also highlighting potential solutions to bridge existing gaps and provide a roadmap for future advancements in AI-driven healthcare.

### 4.1 Data heterogeneity and integration

Biomedical and behavioral research generates highly complex and heterogeneous data from various sources, including clinical records, medical imaging, wearable sensor outputs, and environmental factors and . These datasets are often collected using different protocols, measurement techniques, and devices, leading to inconsistencies and a lack of standardization. Integrating such heterogeneous data sources remains a significant challenge, necessitating novel AI-driven fusion techniques capable of handling missing data, varying resolutions, and conflicting formats. Furthermore, regulatory and ethical constraints, such as data privacy laws (e.g., , ), add another layer of complexity to data sharing and integration. Although these laws are essential for protecting sensitive health information, they also pose challenges for researchers seeking to leverage large-scale, interoperable datasets. To address these challenges, several promising solutions have been introduced. The development of standardized data ontologies can help harmonize diverse biomedical datasets, improving data interoperability across institutions. Federated learning frameworks enable  models to be trained decentrally across multiple data sources without directly sharing sensitive patient data, addressing both privacy and scalability concerns [49], [41]. In addition, self-supervised learning techniques can mitigate the impact of missing or incomplete data, enhancing the robustness of AI-driven biomedical analysis. Privacy-preserving AI techniques, such as differential privacy and secure multi-party computation, further ensure compliance with regulatory requirements while enabling data sharing. The adoption of these strategies can significantly improve the integration and usability of multimodal biomedical datasets for AI applications.

### 4.2 Model interpretability and explainability

The lack of interpretability and explainability in  models remains one of the main challenges in AI-driven biomedical applications. Many  models function as ‘black boxes’, making it difficult for clinicians and researchers to trust AI-generated predictions. In clinical decision-making, interpretability is critical not only for building trust but also for ensuring that AI-generated predictions align with medical knowledge and can be validated by healthcare professionals. Developing  frameworks, such as SHAP [50], LIME [44], and attention mechanisms, that provide transparent decision-making, uncertainty quantification, and human-interpretable justifications is crucial to increase the adoption of  in clinical settings. Regulatory agencies, such as the , are increasingly requiring explainability as key criteria for the approval of AI-driven medical devices, highlighting its importance in real-world applications. Researchers are also exploring hybrid approaches that combine the predictive power of  with the transparency of simpler models, with the aim of balancing accuracy and interpretability. Addressing these challenges requires the development of advanced methodologies and frameworks. Hybrid AI models, which integrate DL with interpretable statistical techniques, offer a balance between predictive performance and transparency. These models leverage the strengths of both approaches, enabling clinicians to understand and trust AI-driven predictions. In addition, uncertainty-aware AI frameworks can quantify confidence levels in predictions, providing clinicians with actionable insights and improving the reliability of AI-driven recommendations. Finally, human-in-the-loop AI systems, where AI models provide interpretable insights while clinicians retain decision-making authority, ensure more ethical and reliable adoption of AI in healthcare. These systems foster collaboration between AI and healthcare professionals, improving both trust and clinical utility.

### 4.3 Real-time processing and computational constraints

Implementing AI-driven biosensing solutions in clinical settings requires addressing challenges related to computational cost and real-time processing. AI-driven biosensing and real-time health monitoring demand high-speed processing and low-latency decision-making. However, many  models are computationally expensive and require significant processing power, making them unsuitable for edge devices or mobile-based implementations. Energy efficiency is another critical consideration, as many edge devices and wearables rely on battery power, requiring  models that minimize energy consumption. Optimizing  algorithms for real-time inference, reducing computational overhead, and leveraging edge computing solutions, such as model quantization, pruning, and federated learning, are essential for practical deployment in resource-constrained settings. Furthermore, scalability remains a significant challenge, as real-time health monitoring systems must handle large volumes of data from multiple users simultaneously. Several approaches have been proposed to address these challenges. Model optimization techniques, such as quantization and pruning, reduce model size and computational requirements, enabling faster inference on resource-limited devices. Federated learning allows  models to be trained decentrally across multiple devices, reducing the need for high-bandwidth data transfers while preserving user privacy [49]. In addition, leveraging edge AI architectures, including lightweight transformer models and hardware-aware neural networks, improves latency and energy efficiency. Finally, cloud-edge hybrid systems dynamically offload complex computations to the cloud while handling time-sensitive tasks locally, ensuring scalable and efficient real-time processing in AI-driven healthcare applications.

### 4.4 Data privacy, security, and ethical considerations

The integration of  with sensitive biomedical data raises critical concerns about privacy, security, and ethical use. Ensuring data anonymization, secure storage, and compliance with regulations such as  and  is essential to maintain patient confidentiality. Data breaches pose a significant risk to patient privacy, necessitating robust cybersecurity measures such as encryption, access controls, and regular security audits. In addition, ethical issues regarding bias in AI models, data ownership, and informed consent must be addressed to prevent disparities in healthcare outcomes. Algorithmic transparency is equally important to ensure that AI-driven decisions can be audited and understood by healthcare providers, patients, and regulators. Compliance with various regulatory frameworks, such as  in the U.S. and  in the , adds complexity to the global deployment of AI-driven biomedical applications. To address these challenges, several strategies have been developed. Privacy-preserving AI techniques [42], such as federated learning [51] and differential privacy [52], [53], enable secure AI model training without exposing raw patient data, ensuring compliance with privacy regulations. Homomorphic encryption and secure multi-party computation allow AI systems to process encrypted biomedical data, minimizing security risks. Implementing , zero-trust security architectures, and real-time anomaly detection can improve cybersecurity and prevent unauthorized access to sensitive health data. Furthermore, standardizing AI regulatory frameworks and establishing cross-border legal interoperability can facilitate the ethical and responsible deployment of AI-driven healthcare applications in different regions.

### 4.5 Generalizability and bias in AI models

 models trained on limited or biased datasets often struggle to generalize across diverse patient populations and clinical environments. Biomedical and behavioral datasets are affected by demographic imbalances, leading to AI-driven decisions that are less reliable for underrepresented groups. Furthermore, many datasets suffer from biases due to the underrepresentation of certain populations, which limits the generalizability of  models in real-world applications. Biased predictive models can lead to misdiagnosis or unequal access to care, disproportionately affecting marginalized populations and exacerbating existing health disparities. To improve the generalizability of the model and reduce bias, it is necessary to develop robust  models that incorporate diverse datasets, domain adaptation techniques, and fairness constraints, such as adversarial debiasing, reweighting, and fairness-aware algorithms. Improving data collection practices to ensure diversity and representativeness is also crucial, as well as the use of fairness-based evaluation metrics, such as equalized odds or demographic parity, to assess  models' performance across different demographic groups. To mitigate bias and improve generalizability, various strategies can be employed. Data augmentation and synthetic data generation techniques, such as  [54] and -based data synthesis [55], can expand training datasets to include underrepresented populations, improving model performance across various groups. Domain adaptation methods, such as  [56], transfer learning, and self-supervised contrastive learning, allow AI models to adapt to new clinical environments while maintaining high performance. Fairness-aware AI techniques, including adversarial debiasing [40], reweighting strategies [57], and fairness constraints, can proactively address bias during model training. Algorithms such as adversarial debiasing and reweighting have been applied to reduce bias in predictive models for healthcare [58], [59]. Beyond model-level solutions, regulatory guidelines should enforce bias audits and fairness evaluations to ensure equitable AI deployment in healthcare applications.

Integrating  with biomedical and behavioral data presents challenges, but also allows innovation that can improve healthcare. Addressing issues such as data heterogeneity, model interpretability, real-time processing, and ethical considerations requires a holistic approach that combines advanced  methods with scalable, low-cost technologies. In the following section, we propose a framework that leverages mobile-based colorimetry and multimodal data fusion to address these challenges, enabling accurate, interpretable, and real-time health monitoring. This framework bridges the gap between laboratory-grade accuracy and real-world applications, making AI-driven healthcare solutions more scalable, reliable, and clinically applicable.

### 4.6 From challenges to framework design

The challenges described in this section, ranging from data heterogeneity and limited model interpretability to computational limitations and algorithmic bias, highlight the need for integrated and practical AI solutions in healthcare. Although existing approaches offer partial solutions, such as XAI methods, privacy-preserving learning, model optimization for edge deployment, or fairness-aware learning algorithms, there is currently no unified framework that integrates mobile-based colorimetric biosensing with multimodal behavioral and clinical data in an interpretable and scalable manner. Our review also identified that mobile-based colorimetry is often evaluated in isolation, without integration into broader predictive frameworks involving contextual data, such as wearable sensor streams, environmental exposures, or electronic health records. Similarly, although multimodal AI has made advances in clinical informatics, its integration with low-cost, real-time sensing technologies for behavioral health remains limited. These findings highlight a critical gap in end-to-end frameworks capable of: (*1*) integrating mobile biosensing with contextual behavioral and clinical data; (*2*) supporting interpretable, real-time AI inference at the edge; and (*3*) enabling scalable, personalized, and privacy-preserving diagnostics. To address this issue, we propose an AI-driven multimodal colorimetric analytics framework that bridges mobile-based biosensing with multimodal data fusion and explainable AI. Section 5 outlines the conceptual structure of this framework and its components, grounded in the insights identified through our review.

## 5 A unified conceptual framework for AI-driven health diagnostics

**Fig. 1:**
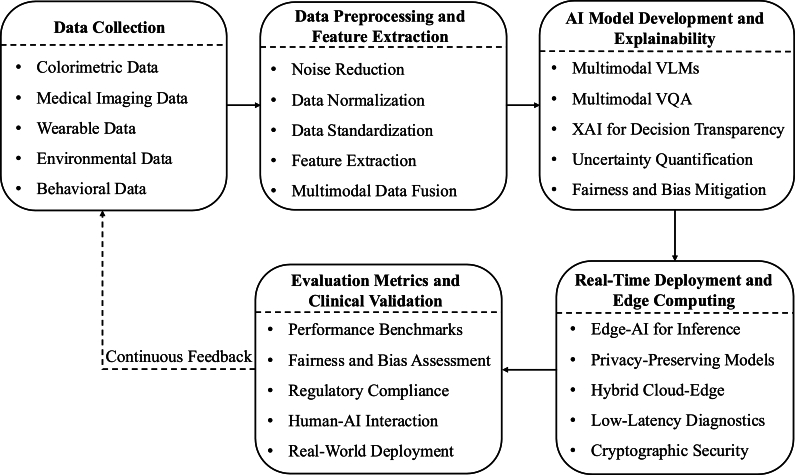
A high-level modular framework for AI-driven multimodal colorimetric analytics. The system integrates mobile biosensing, behavioral and environmental data, multimodal fusion, explainable AI, and edge-compatible deployment to support real-time, interpretable, and privacy-aware diagnostics. Components are designed to interoperate through feedback loops, enabling continuous refinement and adaptation to evolving clinical needs.

**Fig. 2:**
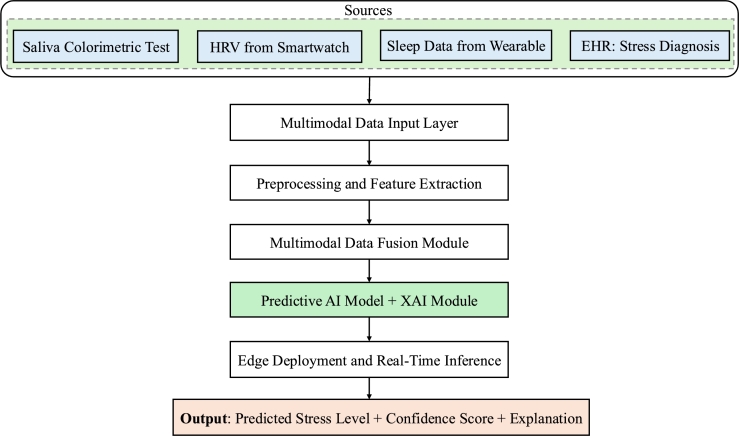
Example workflow for mobile-based stress monitoring using the proposed AI-driven multimodal framework. Saliva colorimetric testing, wearable sensor data (HRV and sleep patterns), and clinical records are integrated through a unified pipeline that involves preprocessing, multimodal data fusion, interpretable AI modeling, and real-time inference on edge devices. The system outputs a predicted stress level with a confidence score and an explanation.

To address the challenges outlined in the previous section, we propose a unified conceptual framework that integrates mobile-based biosensing, multimodal data fusion, behavioral signal modeling, and explainable AI into a cohesive end-to-end pipeline. The proposed framework is designed for decentralized, real-time environments and supports adaptive, privacy-aware deployment at the edge. It consists of five interconnected stages: (*i*) mobile colorimetric biosensing, (*ii*) preprocessing and signal conditioning, (*iii*) multimodal data fusion, (*iv*) interpretable AI modeling, and (*v*) real-time inference. Fig. 1 presents a high-level modular view of the proposed framework, and each component is discussed in detail below. This framework leverages real-time colorimetric analysis,  models, and secure data integration techniques to improve diagnostic accuracy, accessibility in low-resource settings, and support context-aware, personalized healthcare delivery. In the context of behavioral health, it enables the noninvasive monitoring of stress, mental health, and substance use biomarkers. Furthermore, our framework follows a continuous learning and refinement process, where insights from evaluation metrics and clinical validation are used to improve upstream data collection and model generalization. This ensures that the system's performance and reliability are continuously enhanced. By incorporating feedback loops across the pipeline, the framework reduces diagnostic bias and aligns with evolving clinical priorities. This iterative and learning-oriented approach makes the framework both adaptive and scalable. The proposed system includes the following key components:

This framework enables diagnostic systems that deliver not only high predictive accuracy, but also explainability, scalability, and practical deployability in real-world healthcare settings, including low-resource settings. In the following sections, we map recent developments in each of these components to highlight progress, challenges, and opportunities. To make our proposed modular framework more concrete, we present an example workflow for mobile-based stress monitoring (Fig. 2). In this scenario, saliva colorimetric tests, wearable sensor data (e.g., heart rate variability (HRV) and sleep patterns), and clinical records are integrated through multimodal data fusion and interpretable AI modeling. The proposed system enables real-time, privacy-preserving deployment at the edge, providing actionable diagnostic outputs, such as predicted stress levels with associated confidence scores and explanations. Table 5 summarizes the primary data sources and measurement types used in the example workflow, providing concrete examples of how multimodal inputs are collected and integrated.

**Table 5:** Summary of data sources integrated in the mobile-based stress monitoring workflow.

Source	Data Type	Measurement Type	Example
Saliva Colorimetric Test	Biosensor (colorimetric)	Biochemical stress marker (e.g., cortisol level via color change)	Saliva sample, color strip changing color, smartphone image capture
HRV from Smartwatch	Wearable physiological signal	Heart rate variability (biomarker for stress resilience)	Smartwatch or chest strap measuring beat-to-beat variability
Sleep Data from Wearable	Behavioral/physiological data	Sleep duration, sleep quality (stress indicators)	Wearable devices such as Fitbit, Apple Watch, or Oura Ring
EHR Stress Diagnosis	Clinical record	Diagnosis/history of stress-related disorders (ICD-10 codes)	Doctor's notes or diagnostic history stored in EHR

### 5.1 Data collection

The first stage of our proposed framework involves collecting diverse multimodal data to enable robust AI-driven analysis. These data sources range from point-of-care diagnostics to continuous physiological monitoring and structured clinical records. To ensure compliance with privacy regulations and protect sensitive health information, anonymization and encryption of data are typically used during the collection process. These measures are essential to maintain data security and comply with regulatory standards such as  and .

**Mobile-Based Colorimetric Biosensors**. Smartphone cameras and AI algorithms detect visible colorimetric changes in biological and chemical fluids (e.g., blood, saliva, urine), allowing low-cost and scalable biomarker quantification [13], [60]. This approach is particularly useful for early disease detection and real-time health monitoring, especially in resource-limited settings. Recent  methods have demonstrated strong potential to automate the interpretation of mobile-captured colorimetric assays. Studies have applied  and random forest classifiers to colorimetric assay analysis, achieving high accuracy in analyte classification tasks on mobile-based platforms [7], [12]. Our proposed framework builds on these advances by integrating multimodal data streams beyond isolated colorimetric readings. By facilitating remote diagnostics without reliance on centralized laboratory infrastructure, mobile-based colorimetry significantly improves healthcare access. AI models have significantly improved diagnostic accuracy in colorimetric biosensing by automatically extracting spectral and spatial features from colorimetric images and learning complex relationships between biomarker signals [13]. By integrating contextual data, such as environmental conditions, user-specific factors, or metadata, these models also help reduce noise and minimize false positives [61]. , for example, have been widely applied to quantify subtle color transitions that may be imperceptible to the human eye [12]. Attention-based fusion models further improve inference accuracy by prioritizing signal-rich modalities in multimodal data streams [62], [63]. When combined with wearable sensor data or clinical records, AI-driven approaches enable redundancy that enhances robustness and reliability, particularly in resource-limited settings [64].

**Wearable Health Sensors**. Devices such as smartwatches, continuous glucose monitors, and fitness trackers capture real-time physiological signals, including heart rate, oxygen saturation, body temperature, and physical activity [4]. These continuous data streams enable more responsive and individualized health monitoring while supporting early detection and intervention. Wearable sensors also generate longitudinal datasets that allow  models to learn health patterns over time and detect deviations that may indicate emerging conditions.

**Clinical and Medical Imaging Data**. Structured , radiological images (e.g., MRI, CT scans), and histopathology slides provide a detailed patient history and high-dimensional diagnostic insights [5], [1], [65]. These datasets improve AI-driven diagnostics by integrating multimodal clinical information, allowing more accurate disease detection and treatment planning. In addition, medical imaging data can be combined with other modalities to provide a comprehensive view of the health of a patient.

**Environmental and Behavioral Data**.  sensors and digital surveys collect data on external factors such as air quality, sleep patterns, stress levels, and activity levels [6]. These contextual insights enable AI models to better assess the impact of environmental and lifestyle factors on health outcomes. For example, integrating air quality data with respiratory health metrics can help identify environmental triggers for conditions such as asthma. Behavioral data, such as sleep and activity patterns, can also inform personalized interventions for the management of chronic disease.

### 5.2 Data preprocessing and feature extraction

As part of our proposed framework, raw data from biosensors, wearables, and clinical records must be cleaned, standardized, and structured before AI models can process it. This component focuses on ensuring data quality, reliability, and compatibility, enabling robust and interpretable AI-driven analysis. Data preprocessing techniques such as noise reduction, missing data imputation, and normalization are commonly applied to address challenges in data quality and prepare datasets for AI modeling. These techniques are essential for handling the heterogeneity and variability that often occur in multimodal biomedical data.

**Data Standardization and Normalization**. Our approach aligns heterogeneous data from biosensors, wearables, and  into a consistent format to ensure seamless AI processing and interoperability. Standardization techniques, such as z-score normalization and min-max scaling, could be applied to bring all data into a common range, while domain-specific standardized data ontologies and domain adaptation methods, such as transfer learning and , could be used to harmonize data semantics across sources.

**Noise Reduction and Data Augmentation**. Sensor readings and other data streams often contain noise due to environmental factors or device limitations. Denoising filters, such as wavelet transforms and Kalman filters, are employed to eliminate inconsistencies and improve data quality. Furthermore, synthetic data generation techniques, such as  and , may be used to augment datasets, enhancing the robustness and reliability of AI models.

**Handling Missing Data with Self-Supervised Learning**. Given that many real-world biomedical datasets frequently contain missing values due to incomplete records or device malfunctions, our framework employs self-supervised learning and advanced imputation techniques, such as  and matrix factorization, to enable AI models to make reliable predictions even with incomplete data. These methods could ensure that missing data do not compromise the accuracy or generalizability of the models.

**Feature Extraction and Dimensionality Reduction**. Extracting biomarker-specific features from colorimetric images, wearable sensor data, and clinical datasets is a promising direction to improve model interpretability and computational efficiency. Techniques such as , , and autoencoders are commonly used to reduce dimensionality while preserving critical information. Incorporating these methods could minimize computational complexity and improve the performance of downstream  models, particularly in real-time or resource-constrained settings.

**Image Processing and DL**. To extract relevant spectral features from colorimetric biosensing images, we propose to employ . These models effectively capture localized patterns in the imaging data, enabling the accurate detection and quantification of biomarkers. Leveraging  can yield robust and informative representations that support reliable downstream analysis and interpretation.

**Fig. 3:**
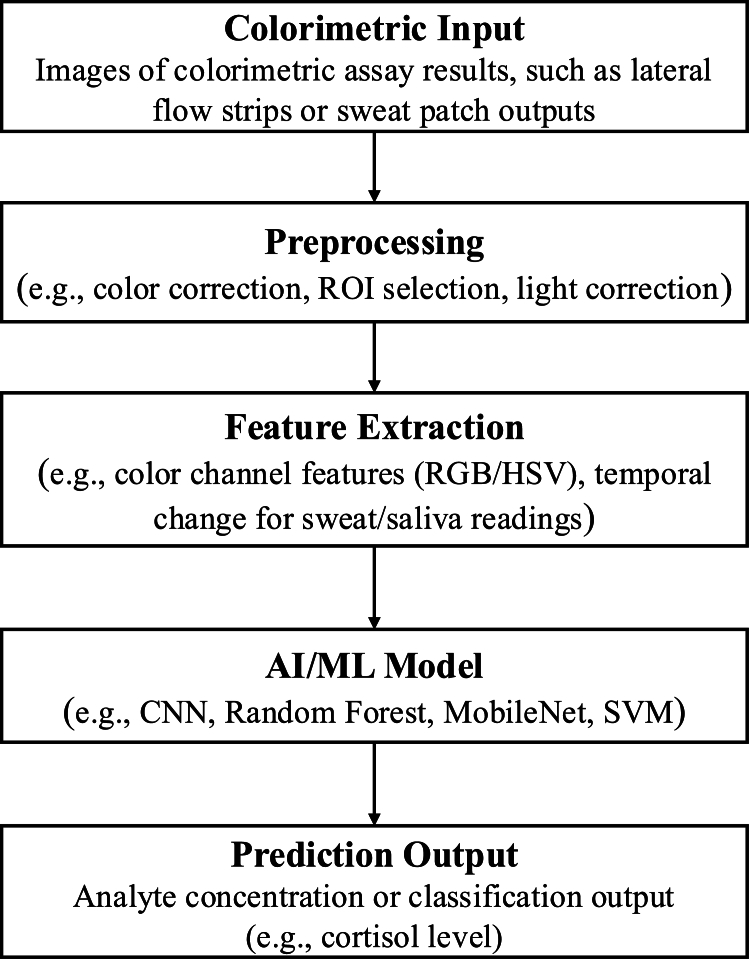
Generalized AI pipeline for colorimetric biosensing. Multimodal colorimetric inputs are processed through key stages including preprocessing (e.g., color correction and normalization), feature extraction (e.g., RGB/HSV channel analysis), and AI modeling (e.g., CNNs, SVMs, MobileNet). The output yields a predicted analyte concentration or classification.

Fig. 3 provides a detailed view of how raw colorimetric signals from wearable or smartphone-integrated biosensors are processed and analyzed to produce actionable predictions. The pipeline begins with image-based or photonic signal acquisition, followed by preprocessing steps such as region-of-interest (ROI) detection and color normalization. Feature representations (e.g., RGB/HSV channels) are then extracted and passed through AI/ML models such as CNNs, MobileNet, random forests, or regression algorithms for the prediction of analyte concentration or biomarker classification. This modular and interpretable workflow complements the broader system-level view shown in Fig. 1 by detailing the signal-level transformations involved in real-time biosensing applications. The pipeline shows the modularity and interpretability features that support our proposed framework. Building on this pipeline, future work should focus on developing lightweight and interpretable models that maintain high accuracy under real-world constraints, including varying lighting, limited training data, and deployment on edge devices.

**Multimodal Fusion Models**. For robust decision-making in biomedical research, processed biosensor outputs, such as colorimetric readings, are integrated with other data streams, including wearable-derived physiological signals, self-reported behavioral inputs, medical imaging, and clinical history. This integration is achieved using advanced multimodal fusion models that employ techniques such as feature-level concatenation, cross-modal attention, and graph-based integration. Transformer-based architectures and  are particularly effective in capturing complex relationships and dependencies across these heterogeneous data sources [66]. By enabling seamless fusion of diverse modalities, these models improve predictive performance, increase generalizability, and support more comprehensive and individualized diagnostics.

### 5.3 AI model development and explainability

The integration of advanced AI architectures is critical for effective biomedical and behavioral analytics. This component of our framework focuses on training AI models to analyze multimodal biomedical data, provide interpretable insights, and ensure bias-free decision-making. By incorporating  and , our framework will improve colorimetric biosensing and multimodal medical analysis.  will process both visual data (e.g., colorimetric images) and textual data (e.g., clinical notes), enabling context-aware analysis and improving the interpretability and usability of AI-driven insights. This approach could enable interactive, explainable, and context-aware AI-driven insights, ensuring transparency, fairness, and usability in clinical settings.

**Multimodal Predictive AI Models**. Supervised ML and DL models, such as [67], Random Forest, and attention-based Transformers [68], are commonly used to predict the likelihood of the disease based on multimodal data, including colorimetric biomarkers, clinical text, and wearable sensor streams. These models capture complex patterns and temporal dependencies across various data sources, enabling accurate and reliable predictions for clinical decision-making. Advanced techniques such as contrastive learning [69], [70] and hybrid neural networks are also being explored to integrate data from colorimetric biosensors, wearable devices, and clinical records, ensuring robust and accurate analysis of heterogeneous data.

**Multimodal**. To enable cross-modal reasoning and analysis, state-of-the-art [71], such as BLIP [72], CLIP [73], OpenCLIP [74], Flamingo [75], LLaVA [76], and LLaVA-Med [77] are increasingly being used to jointly process colorimetric images, clinical text reports, and additional sources of biomedical data. These models bridge the gap between visual and textual data, facilitating context-aware analysis and improving the interpretability of AI-driven insights. By integrating , researchers can fuse colorimetric biosensor images [39], wearable sensor readings, and clinical text data [78], generating textual descriptions of biosensor outputs and correlating them with medical records. This capability improves the contextual understanding of diagnostic information, allowing clinicians to interpret complex data more effectively. Attention-based architectures [68] are also commonly employed to capture complex relationships and dependencies between heterogeneous data sources, ensuring seamless multimodal fusion.

**Multimodal**. Multimodal  systems are increasingly being incorporated into AI frameworks, allowing clinicians to interact with AI models by asking questions about visual data (e.g., “*What does this color change indicate?*” or “*Does this colorimetric test indicate a high glucose level?*”) [79], [80]. These systems analyze input images and structured textual data to provide accurate and explainable answers, improving diagnostic decision-making and the integration of clinical workflows. By leveraging , clinicians can obtain rational responses based on biomedical images, colorimetric sensor data, and clinical knowledge, improving their ability to interpret complex diagnostic information. Multimodal  improves the usability of AI systems in real-world clinical settings by making them more interactive, intuitive, and supportive of clinical decision-making.

**for Decision Transparency**. To ensure trust and usability in clinical settings,  techniques such as SHAP [50], LIME [44], and attention-based visualization mechanisms are increasingly employed to explain model predictions. These methods provide interpretable explanations that allow clinicians to understand and validate the reasoning behind AI-driven decisions. In parallel, diagnostic models are being designed with inherent interpretability, including decision trees, attention-based transformers, and prototype-based or inherently explainable neural networks. By combining these models with XAI tools and , researchers can generate human-readable explanations, such as textual summaries of diagnostic outcomes, rather than purely numerical outputs. However, challenges such as ensuring the accuracy of explanations, scaling XAI techniques to complex architectures, and aligning interpretability techniques with clinical workflows remain areas of active research. This approach can improve transparency and trust in AI-driven diagnostics by making predictions explainable and aligned with evidence-based clinical practice.

**Fairness-Aware and Bias Mitigation Techniques**. Fairness-aware algorithms and diverse training datasets are increasingly being incorporated into AI frameworks to mitigate bias and ensure equitable healthcare outcomes across demographic groups. Techniques such as adversarial debiasing, reweighting, and fairness-aware learning are commonly employed to minimize biases in AI decision-making. These approaches help ensure that AI systems provide unbiased and equitable predictions, promoting fairness in healthcare delivery.

**Uncertainty Quantification and Confidence Estimation**. To improve clinical decision-making, AI frameworks are increasingly designed to provide uncertainty scores alongside predictions, rather than binary decisions. This allows clinicians to assess the confidence level of the AI system in its predictions. Techniques such as Bayesian neural networks and Monte Carlo dropout are commonly used to quantify uncertainty, enabling clinicians to make informed decisions based on the reliability of AI-generated insights.

### 5.4 Real-time deployment and edge computing

To be practically useful in healthcare settings, AI models need to be efficient, scalable, and capable of running in real-time. This component of the framework focuses on delivering low-latency inference, privacy-preserving computation, and seamless integration of edge and cloud computing solutions. Advanced techniques are increasingly being used to provide real-time diagnostics, while also keeping data secure and aligned with regulatory requirements.

**Edge AI for On-Device Inference**. Lightweight AI models optimized for mobile and embedded hardware are increasingly being used to enable real-time inference on the device [81]. These models are able to process biosensor outputs and wearable data locally, providing healthcare professionals with immediate feedback on biomarker analysis. To support deployment in resource-constrained environments, the entire pipeline can be optimized using techniques such as model quantization, pruning, and federated learning. By executing inference directly on edge devices, this approach reduces latency, preserves data privacy, and ensures rapid and context-aware decision-making in time-sensitive clinical scenarios.

**Privacy-Preserving AI**. To address privacy concerns and comply with regulations such as  and , federated learning [49] is being increasingly utilized. This approach enables secure AI training on multiple hospital networks or mobile devices without centralizing or exposing sensitive patient data [41]. By training models in a decentralized fashion, federated learning ensures data privacy while maintaining the accuracy and generalizability of AI-driven insights.

**Cloud-Edge Hybrid Deployment**. A hybrid cloud-edge deployment strategy is increasingly being adopted to balance computational efficiency and real-time performance. AI-driven predictions that require high computational resources, such as complex multimodal analysis, are typically offloaded to cloud servers. However, latency-sensitive tasks, such as real-time biomarker monitoring, are handled locally on edge devices. This hybrid approach ensures scalability and responsiveness in various healthcare settings.

**Low-Latency Diagnostics**. Optimized AI architectures, including quantized neural networks [82], [83] and model distillation techniques [84], [85], are increasingly being employed to ensure the rapid processing of biomedical data. These optimizations enable healthcare professionals to receive instant feedback on biomarker analysis, enhancing the usability of AI frameworks in real-world clinical workflows. Low-latency diagnostics are critical for time-sensitive applications, such as early disease detection and emergency response.

**Advanced Cryptographic Techniques**. To further improve data privacy, advanced cryptographic techniques, such as secure multi-party computation [86], [87] and homomorphic encryption [88], are being increasingly incorporated into AI frameworks. These methods allow AI models to analyze encrypted biomedical data without decrypting it, ensuring end-to-end privacy and security. By leveraging these techniques, we can enable secure collaboration across institutions while protecting sensitive patient information.

### 5.5 Evaluation metrics and clinical validation

Before deploying AI models in real-world healthcare applications, their performance must be rigorously validated using standardized evaluation metrics and clinical trials. This final component of our proposed framework focuses on ensuring the reliability, fairness, and regulatory compliance of AI-driven diagnostics, as well as their feasibility for real-world deployment.

**Performance Benchmarks**. AI models are typically evaluated using standard metrics such as accuracy, precision, recall, ROC-AUC, and F1-score. These metrics ensure that the models provide reliable and accurate predictions for clinical decision-making. For tasks involving , such as , additional metrics such as ROUGE, BERTScore, BLEU, and CIDEr [89] are commonly used to assess the quality of language-grounded responses. If VQA is used, the VQA Score is also included to evaluate the accuracy and relevance of answers generated by the underlying AI system.

**Fairness and Bias Assessment**. To ensure ethical AI decision-making, rigorous fairness evaluations are increasingly being incorporated into AI frameworks. Metrics such as demographic parity, equalized odds, and disparate impact analysis are commonly used to detect and mitigate biases in AI models. In addition, techniques such as adversarial debiasing and fairness-aware learning are employed to ensure equitable outcomes across diverse demographic groups. However, challenges such as defining fairness in domain-specific contexts, addressing intersectional biases, and ensuring scalability in diverse healthcare settings remain areas of active research. These measures promote trust and fairness in AI-driven healthcare applications, offering significant potential to advance equitable healthcare solutions.

**Regulatory Compliance and Clinical Trials**. AI-driven diagnostics must undergo rigorous clinical validation studies to ensure their safety and efficacy before deployment. The framework is designed to comply with regulatory standards such as , , , etc. Clinical trials typically involve comparing AI-generated predictions with gold-standard medical diagnoses to validate their accuracy and reliability. This step is critical to obtain regulatory approval and ensure patient safety.

**Human-AI Interaction Assessment**. For models incorporating  and , human-AI interaction is typically evaluated by clinical experts. This assessment focuses on the model's ability to generate clinically relevant explanations and provide actionable insights. Human-in-the-loop evaluations ensure that the AI system is interpretable, user-friendly, and aligned with clinical workflows.

**Real-World Testing and Deployment Feasibility**. To assess scalability, generalizability, and robustness, AI frameworks are typically tested in various healthcare settings. Real-world testing involves deploying the system in clinical environments to evaluate its performance under practical conditions. This step ensures that the framework is feasible for full deployment and can adapt to the complexities of real-world healthcare scenarios.

**Fig. 4:**
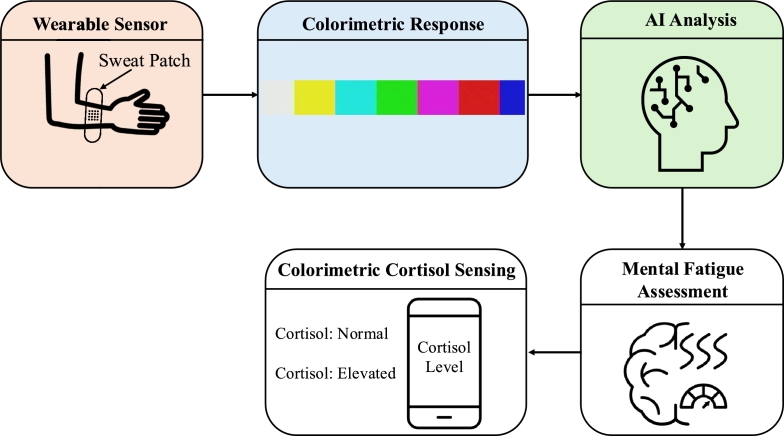
Behavioral health use case enabled by AI-driven colorimetric biosensing. A wearable sweat patch captures colorimetric signals (e.g., cortisol levels), which are processed using AI models to assess mental fatigue. The results, interpreted on a mobile interface, offer real-time and privacy-aware insights for stress-related health monitoring.

To show how AI-powered colorimetric biosensing can be applied to behavioral health, we present a representative use case in Fig. 4. This scenario demonstrates how a wearable patch can be used to monitor stress-related biomarkers such as cortisol through colorimetric analysis. The signal is interpreted by AI algorithms and displayed on a mobile interface, enabling real-time assessment of mental fatigue. This end-to-end workflow demonstrates how personalized, non-invasive, and scalable tools can support mental health monitoring in daily life. Moving forward, this scenario shows the need for further validation of AI-powered colorimetric tools in real-world settings and their integration with privacy-preserving, longitudinal monitoring strategies for mental health support.

## 6 Conclusion and future work

The integration of AI-driven multimodal colorimetric analytics has the potential to revolutionize biomedical and behavioral health research by improving diagnostic accuracy, improving accessibility, and supporting personalized medicine. By combining mobile-based biosensors, AI-enabled feature extraction, multimodal data fusion, and real-time deployment, this framework offers a scalable and cost-effective solution for disease detection and health monitoring. Incorporating XAI techniques and fairness-aware algorithms ensures the transparency, reliability, and ethical use of AI-driven healthcare solutions. However, several challenges remain, including improving the generalizability of AI models across diverse populations, addressing data heterogeneity, and ensuring regulatory compliance for widespread clinical adoption. Future research directions include refining AI models through adaptive learning techniques, expanding dataset diversity to mitigate biases, and exploring privacy-preserving approaches such as federated learning and secure multi-party computation. Furthermore, the integration of VLMs and Visual QA can further improve model interpretability and usability in clinical settings, allowing healthcare professionals to interact with AI-driven systems and improving adoption in real-world workflows. Establishing standardized evaluation benchmarks for colorimetric biosensing platforms could also help to allow more consistent performance reporting and fair comparisons across studies. Designing adaptable AI architectures that operate reliably across device types would enhance scalability, deployment flexibility, and generalizability, an aim already supported by the modular structure of our proposed framework. By addressing these research gaps, future advancements in AI-driven multimodal colorimetric analytics can enable the development of more robust, interpretable, and scalable AI applications in healthcare. Such innovations could reshape the way diagnostics are delivered, making them faster, more accessible, and more equitable, especially in low-resource environments.

## CRediT authorship contribution statement

**Desta Haileselassie Hagos:** Writing – review & editing, Writing – original draft, Validation, Methodology, Investigation, Formal analysis, Data curation, Conceptualization. **Saurav Keshari Aryal:** Writing – review & editing, Validation, Resources, Project administration, Funding acquisition, Conceptualization. **Patrick Ymele-Leki:** Writing – review & editing, Validation, Resources, Project administration, Investigation, Funding acquisition. **Legand L. Burge:** Validation, Supervision, Resources, Project administration, Funding acquisition, Conceptualization.

## Declaration of Competing Interest

None.
